# Suppressive compost: snake oil remedies or achievable goal?

**DOI:** 10.1128/aem.02019-25

**Published:** 2025-12-03

**Authors:** Giuliano Bonanomi, Mohamed Idbella

**Affiliations:** 1 Department of Agricultural Sciences, University of Naples Federico II 9307, Portici, Italy; 2 AgroBioSciences (AgBS) program, College of Agriculture and Environmental Sciences, Mohammed VI Polytechnic University 479571, Ben Guerir, Morocco; Michigan State University, East Lansing, Michigan, USA

**Keywords:** compost, disease suppression

## Abstract

In recent decades, many researchers have aimed to create suppressive composts and determine the factors that distinguish them from non-suppressive ones. The research conducted by A. Logo, B. Boppré, J. Fuchs, M. Maurhofer, et al. (Appl Environ Microbiol 91:e01100-25, 2025, https://doi.org/10.1128/aem.01100-25) represents a significant advancement in understanding the microbiological bases of this phenomenon, as it evaluates the suppressiveness of 37 composts across three different pathosystems. The authors successfully identified certain bacterial and fungal taxa that could potentially act as indicators of specific disease suppressiveness.

## COMMENTARY

Composting is a biological process that can convert organic materials that cannot be directly used in agriculture into a stable and non-phytotoxic humified substance within a few weeks, which can aid in plant growth. A critical feature of compost is its ability to suppress pathogens, meaning it can reduce disease incidence and severity, although this does not necessarily imply the elimination of the pathogen population. While natural suppression of diseases has long been recognized in composts (1), it is also observed in soils (2), peat (3), and nitrogen-rich organic amendments (4); it remains a challenging ecosystem function to fully understand. The suppressive capacity of compost can greatly vary depending on the type of feedstock used, the conditions under which it is processed, and the length of the maturation period, allowing it to be highly effective against various soilborne and foliar pathogens. Nevertheless, in some instances, compost may fail to limit the spread of pathogens, and in more uncommon cases, it can even contribute to an increase in the occurrence and severity of diseases (5). The significant fluctuations in disease suppression are particularly evident with pathogens known for their strong saprophytic abilities, such as *Rhizoctonia solani*, *Fusarium oxysporum*, and *Globisporangium ultimum*. These pathogens can compete with beneficial microbes and utilize readily available organic carbon, thereby enhancing their potential for infection and may cause more severe attacks on plants. It is, therefore, crucial to distinguish between composts that effectively suppress pathogens and those that do not, to manage this characteristic effectively across various cultivation systems. This differentiation is a vital step in dispelling the notion that compost suppressiveness is an unpredictable quality, which some researchers have likened to snake oil remedies.

In the last 50 years, numerous experimental studies have sought to create suppressive composts and determine the factors that can differentiate them from non-suppressive ones. Research comparing various composts derived from different feedstocks and levels of maturity has proved particularly productive. Termorshuizen et al. (6) examined the suppressiveness of 18 composts across 7 pathosystems, observing that disease suppression occurred in 54% of instances, while just 3% exhibited significant disease enhancement, with responses being highly species-specific. Following a similar methodology, Logo et al. (7) assessed 37 composts across three pathosystems, including *R. solani*, which is notoriously challenging to manage with organic amendments (5). As anticipated, the authors noted considerable variability in suppressiveness among composts, both within single plant–pathogen interactions and, more crucially, across different pathosystems. This underscores the challenges in developing and identifying composts with multisuppressive capabilities, that is, those that can effectively manage pathogens with varying functional characteristics (8). From this viewpoint, pinpointing predictive parameters for suppressivity has proven to be an intricate task, despite hundreds of studies conducted over the past 80 years (9).

The complexity of compost suppressivity arises from a combination of chemical properties and microbiome functionality. The substantial biological foundation of suppressivity is evident through the gradual loss of this trait after exposure to increasingly intense heat treatments (10). A meta-analysis performed prior to the transformational impact of metagenomics on microbiome studies, compost, and suppressiveness revealed only weak correlations, with the most promising indicators being the rate of fluorescein diacetate hydrolysis, soil respiration, total microbial biomass, and the population density of fluorescent *Pseudomonas* and *Trichoderma*. The study by Logo et al. (7) reinforces the inconsistency of fundamental chemical parameters in forecasting suppressivity while making strides toward a deeper comprehension of the microbiological foundations of the phenomenon. By contrasting highly suppressive composts with non-suppressive ones, researchers have been able to identify specific bacterial and fungal taxa that may serve as potential indicators of targeted suppressiveness for each plant–pathogen interaction.

It is crucial to keep in mind that while identifying and researching suppressive taxa is essential, it alone does not suffice for creating suppressive composts. Recent advancements in microbiome research have enabled us to uncover the diversity and functionality of microbial components in remarkable detail (11, 12), but they may have inadvertently shifted researchers' focus away from another critical element: the organic fraction of compost. Hoitink and Boehm (13) have made it clear that disease suppression relies on the substrate, meaning that microorganisms perform functions, but they need a suitable organic substrate to be effective. The strong link between the microbiome and substrate is well recognized in the human gut microbiome, where a diet filled with fiber and low in simple sugars and fats is deemed essential for maintaining a healthy microbiome (14). This concept should also be re-emphasized in the context of soil suppression, using methods that can detail the functional profile of organic matter, such as ^13^C CPAMS NMR spectroscopy, and going beyond basic metrics like the C/N ratio, pH, and nutrient levels, which fail to predict the ability of various organic fractions to promote beneficial microbiomes (15). In summary, it is vital to provide an appropriate substrate that encourages suppressive bacteria and fungi while avoiding the support of pathogens, particularly those capable of saprophytic activity.

In conclusion, it is important to note that the composting process is primarily managed by private firms that face various limitations, including the accessibility of organic feedstocks and the timing of the process, which inevitably affects the economic feasibility of their operations. In this context, producing suppressive compost can be a significant advantage, a target that can be reached, for instance, by employing currently proven methods such as inoculating with beneficial microorganisms at the conclusion of the thermophilic stage (16), or by combining certain organic materials like biochar that can serve as a medium for beneficial microbes and diminish the availability of easily digestible carbon, thus hindering pathogens with saprophytic capabilities (17, 18). Considering what has been discussed, creating suppressive compost seems to be a feasible objective, with the potential for use in various cultivation systems to reduce or even substitute fungicides and fumigants.
